# Nitrogen-Doped CuO@CuS Core–Shell Structure for Highly Efficient Catalytic OER Application

**DOI:** 10.3390/nano13243160

**Published:** 2023-12-17

**Authors:** Abu Talha Aqueel Ahmed, Abu Saad Ansari, Vijaya Gopalan Sree, Atanu Jana, Abhishek Meena, Sankar Sekar, Sangeun Cho, Hyungsang Kim, Hyunsik Im

**Affiliations:** 1 Division of Physics and Semiconductor Science, Dongguk University, Seoul 04620, Republic of Korea; abutalha.aa@dongguk.edu (A.T.A.A.); sreevg@dgu.ac.kr (V.G.S.); atanujana@dongguk.edu (A.J.); abhishek@dgu.ac.kr (A.M.); sanssekar@dongguk.edu (S.S.); sangeun.c@dongguk.edu (S.C.); hskim@dongguk.edu (H.K.); 2 Center of Excellence Applied Nanotechnology, Nano Center Indonesia Research Institute, Banten 15314, Indonesia; saad@nano.or.id; 3 Quantum-Functional Semiconductor Research Center, Dongguk University-Seoul, Seoul 04620, Republic of Korea

**Keywords:** water electrolysis, CuO@CuS, oxygen evolution reaction, nitrogenation, hydrothermal growth

## Abstract

Water electrolysis is a highly efficient route to produce ideally clean H_2_ fuel with excellent energy conversion efficiency and high gravimetric energy density, without producing carbon traces, unlike steam methane reforming, and it resolves the issues of environmental contamination via replacing the conventional fossil fuel. Particular importance lies in the advancement of highly effective non-precious catalysts for the oxygen evolution reaction (OER). The electrocatalytic activity of an active catalyst mainly depends on the material conductivity, accessible catalytically active sites, and intrinsic OER reaction kinetics, which can be tuned via introducing N heteroatoms in the catalyst structure. Herein, the efficacious nitrogenation of CuS was accomplished, synthesized using a hydrothermal procedure, and characterized for its electrocatalytic activity towards OER. The nitrogen-doped CuO@CuS (N,CuO@CuS) electrocatalyst exhibited superior OER activity compared to pristine CuS (268 and 602 mV), achieving a low overpotential of 240 and 392 mV at a current density of 10 and 100 mA/cm^2^, respectively, ascribed to the favorable electronic structural modification triggered by nitrogen incorporation. The N,CuO@CuS also exhibits excellent endurance under varied current rates and a static potential response over 25 h with stability measured at 10 and 100 mA/cm^2^.

## 1. Introduction

Fossil fuel technology has been the major source of energy for a long time, but it comes with significant ecological, resource, and efficiency drawbacks [1]. The dynamic change in energy needs with rapidly developing technology has attracted significant attention with regard to the environmental catastrophe caused by the excess use of traditional fossil fuels [2,3]. In this regard, the production of renewable H_2_ fuel offers a more sustainable and eco-friendlier alternative with the potential to address many of these drawbacks, especially when it is produced through the electrolysis of Earth-abundant water (2H_2_O → 2H_2_ + O_2_) [4]. Water electrolysis is categorized into two simultaneous half-reactions, namely the oxygen evolution reaction (OER; 2H_2_O → 4H^+^ + 4e^−^ + O_2_) and the hydrogen evolution reaction (HER; 4H^+^ + 4e^−^ → 2H_2_) [5]. Although H_2_ production from water electrolysis is comparatively easy, O_2_ production is still a bottleneck due to its sluggish reaction kinetics, which deteriorates the overall electrolyzer cell’s efficiency [6]. The development of energy-efficient, inexpensive, and stable OER catalysts is crucial to ensure that water electrolysis becomes a feasible and scalable energy conversion technology [7]. This need has inspired extensive research efforts to create catalyst materials composed solely of abundant and inexpensive elements [7,8].

Due to their high activity at a wide pH range, Ru/Ir-based catalysts are still the benchmarks for OER; however, the high cost and scarcity of the precursors hinder their large-scale application [9]. In recent years, significant interest has been directed towards the first-row transition metal (M)-based oxyhydroxide, oxide, phosphide, chalcogenide, and carbide materials to pursue highly efficient catalysts [10,11]. Specifically, CuS-based catalysts for OER have gained considerable attention due to their cost-effectiveness, abundance, and excellent redox properties and, therefore, have been examined as OER catalysts for water electrolysis applications [12,13]. However, the scalable application of CuS catalysts is restricted due to their poor overpotential at a higher current rate and degraded performance over time, caused by the sluggish OER kinetics, which is linked to the surface oxidation and corrosion of metal sulfides in an alkaline medium [14]. These issues can be overcome by tuning the electronic structure of the active catalyst by introducing heterogeneous N atoms through a doping process and forming a core–shell structure [15]. Generally, M-N bonding leads to better electrical conductivity compared to M-S bonding due to the higher electronegativity of N than S, and nitrogen has a promising electron-withdrawing ability from adjacent Cu in the catalyst structure through donor/acceptor interaction, leading to intrinsic alterations in the electronic configurations of Cu ions within the catalyst material [16].

**Figure 1:**
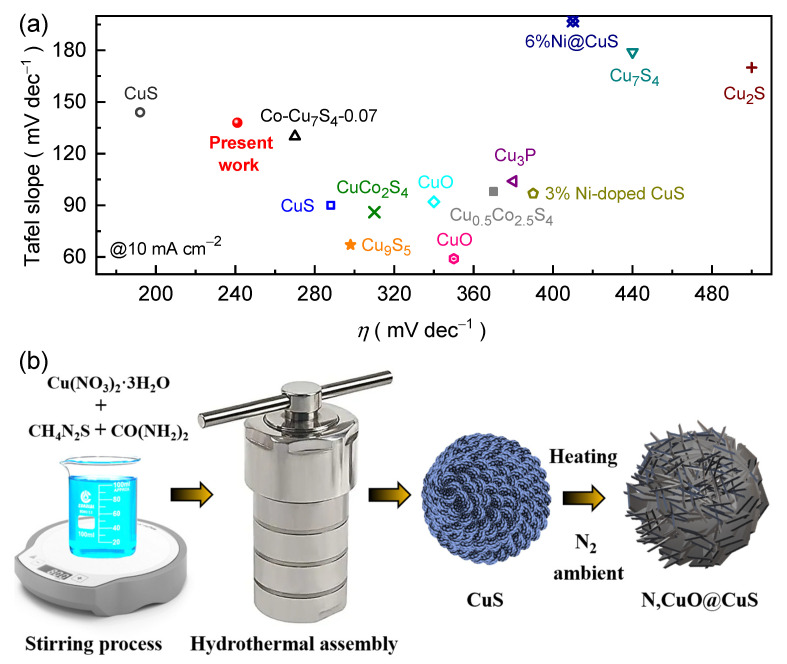
(**a**) Comparative overpotential versus Tafel plots for various Cu-based metal chalcogenide catalysts reported in 1 M KOH electrolyte medium and our N,CuO@CuS catalyst at 10 mA cm^−2^. (**b**) Schematic representation of synthesis procedure for the fabrication of CuS and N,CuO@CuS catalyst. Notably, the catalysts name and the associated symbol colour are identical in Figure 1a [11,17,18,19,20,21,22,23].

Owing to the above fundamental key concept, herein, we have synthesized an N-doped CuO@CuS (N,CuO@CuS) core–shell structure on a macroporous Ni foam (NF) substrate via a hydrothermal and post-nitrogenation process. The electrochemical studies reveal that introducing the heteroatom (N) enhances both the electrical conductivity and catalytically active sites, which leads in turn to the higher intrinsic reaction activity of the proposed catalyst. Dramatically, in contrast to the as-prepared CuS catalyst, the N,CuO@CuS core–shell catalyst exhibits exceptional characteristics in alkaline KOH medium and demonstrates a reduced overpotential (240 mV), a more modest Tafel slope (138 mV dec^−1^), and higher normalized *J_ECSA_* values. The N,CuO@CuS core–shell catalyst also exhibits remarkable endurance with long-term stability (25 h at 10, 50, and 100 mA cm^−2^) after an insignificant change in the potential due to partial electrooxidation during initial testing in an alkaline electrolyte. In addition, the N,CuO@CuS core–shell catalyst maintains the smallest voltage response compared to the CuS catalyst, even with various chalcogenide-based catalysts (Figure 1a), and the obtained results are in an adequate, tolerable range (Figure S1) [11,17,18,19,20,21,22,23].

## 2. Materials and Methods

### 2.1. Materials

All the analytical-grade chemical reagents mentioned were employed in the experiment without undergoing additional purification processes. Thiourea (CH_4_N_2_S), copper nitrate trihydrate (Cu(NO_3_)_2_·3H_2_O), urea (CH_4_N_2_O), potassium hydroxide (KOH), hydrochloric acid (HCl), and ethanol (CH_3_CH_2_OH) were procured from Sigma Aldrich (Merck, St. Louis, MO, USA). The macroporous three-dimensional (3D) NF substrate (used electrode size: 1 × 5 cm^2^) was provided by Alantum (Seoul, Republic of Korea).

### 2.2. Synthesis of CuS and N,CuO@CuS

The CuS film was initially synthesized via a hydrothermal method and subsequently treated in a nitrogen atmosphere to obtain the desired N,CuO@CuS core–shell structure. Figure 1b is a schematic illustration of the fabrication process of the CuS and N,CuO@CuS catalysts with embossed and flake-like textured surfaces, respectively. In a typical procedure, CH_4_N_2_S (50 mmol) and Cu(NO_3_)_2_·3H_2_O (5 mmol) were dissolved in water in a glass beaker containing CO(NH_2_)_2_ (30 mmol). The mixture was kept under stirring for about 30 min at room temperature (R.T.). Thereafter, the NF (exposed area: 1 × 1 cm^2^) and solution were placed in a Teflon-lined autoclave vessel. The assembly was kept in a furnace and the hydrothermal reaction proceeded for 12 h at 160 °C. After natural cooling, the deposited electrode film was taken out, washed with a large amount of deionized water and ethanol, and dried overnight in a vacuum at 80 °C. In the following step, the CuS electrode film was kept in a sealed quartz tube in a tubular furnace. After vacuuming and purging with nitrogen for an hour to remove air, the furnace was heated to 2 h at 350 °C and then cooled to R.T. The resulting N,CuO@CuS film was utilized as the catalyst electrode for the electrochemical OER test.

### 2.3. Material Characterization

The material structure and crystallinity of the proposed catalyst electrode films were analyzed by the X-ray diffraction (XRD) measurement technique using a Rigaku Smartlab instrument, Tokyo, Japan. The XRD spectra were obtained by utilizing CuKα radiation with the incident wavelength (λ) of 1.54056 Å and covering a spectral angle (2θ) range of 20 to 80° at a scanning rate of 2° min^−1^. The XRD instrument was operated at an applied current and voltage of 30 mA and of 40 kV, respectively. The elemental bonds and material fingerprints were examined using Raman spectra analysis using a LabRam Armis instrument with a 514 nm Ar ion laser beam (model: Horiba Jobin Yvon, Kyoto, Japan). The material morphology and composition were assessed using field emission scanning electron microscopy (FESEM) and energy-dispersive spectroscopy (EDS) with a JSM-6701F instrument (model: S-4700, Hitachi, Japan). The EDS spectra were recorded at the low FESEM image magnification of ×5000 and the instrument was operated at 15 kV. X-ray photoelectron spectroscopy (XPS) was employed to assess the oxidation states of the constituent elements, with elemental binding energies calibrated against the chamber’s contaminated carbon (C 1s at 284.33 eV). The XPS spectra were recorded using a PHI 5000 VersaProbe instrument (model: ULVAC PHI, Kanagawa, Japan). The best-fit model for all the acquired individual narrow spectra involved the utilization of Gaussian curve fitting.

### 2.4. Electrochemical OER testing

The electrochemical measurements for catalytic OER application were performed on an electrochemical workstation (AMETEK, VersaSTAT instrument, Berwyn, IL, USA). The three-electrode cell included Pt foil as the counter electrode and a saturated KCl-filled reference electrode (saturated calomel electrode; SCE), whereas the CuS and N,CuO@CuS films served as the working electrodes, 1 M KOH was used as the electrolyte to examine all the electrochemical characteristic properties, and the potentials (V) vs. RHE were converted from V vs. SCE using the following equation:

E_RHE_ = E°_SCE_ + (pH × 0.059) + E_SCE_, (1)

where E_SCE_, E_RHE_, and E°_SCE_ are the voltage in SCE scale, potential in RHE scale, and SCE’s standard potential at R.T., respectively. The linear-scan voltammetry (LSV) curves were measured in a voltage window range between 0.0 and 1.0 V (vs. SCE) to understand the voltage responses of the catalysts, and the measured LSV curves were *JR*-corrected to estimate the overpotential (*ɳ*) values at the driven current densities as follows:

*ɳ* = E_RHE_ (*JR*-corrected) − 1.23, (2)

E_RHE_ (*JR*-corrected) = E_RHE_ − (*J* × *Rs*), (3)

The electrochemically active surface area (*ECSA*) depicts the accessible catalytically active sites of an active catalyst material, which can be obtained by estimating the electrochemical double-layer capacitance (*C_DL_*) at various scan rates (*v*) in the non-Faradaic CV curve region between 0.00 and 0.10 V (vs. SCE), using the following equations:

*C_DL_* = *J_DL_*/*v*, (4)

*ECSA* = *C_DL_*/*C_E_*, (5)

where *J_DL_* is the non-Faradaic region current density and *C_E_* (for KOH electrolyte, the value of *C_E_* is 0.04 mF cm^−2^) is the electrolyte solution’s capacitance. *C_DL_* is simply the slope of the “*J_DL_* versus *v*” plot (i.e., Equation (4)), and its values were calculated at a fixed potential of 0.05 V (vs. SCE) from the scan rate-dependent CV curves. Notably, the positive and negative slopes (i.e., *C_DL_* value) are associated with positive and negative linear charging current densities, respectively, which are due to the non-Faradaic CV sweeps, respectively. In addition, the electrochemical impedance spectroscopy (EIS) technique was carried out in a wide frequency range (0.1 and 10 kHz) to study the fundamental electrochemical charge transfer behavior of the catalysts. All of the impedance curves were recorded at a biasing potential of 0.4 V and 10 mV amplitude of the applied AC signal.

## 3. Results and Discussion

### 3.1. Morphological and Compositional Properties

**Figure 2:**
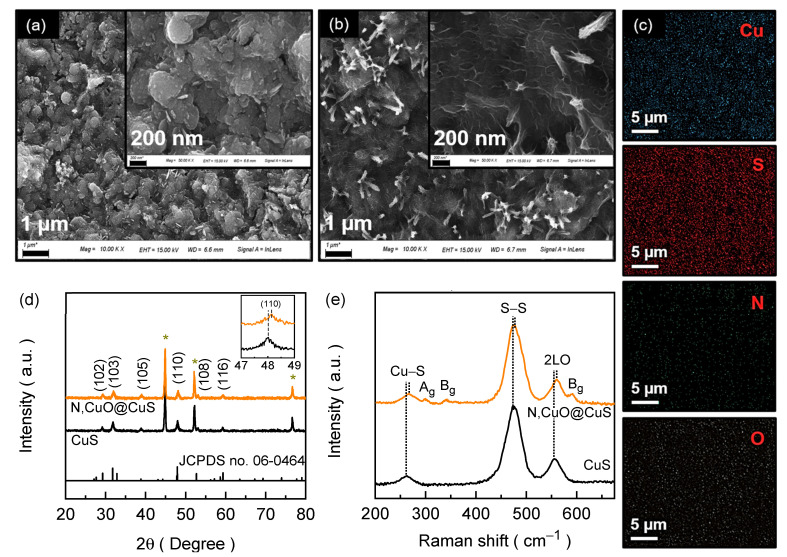
FESEM images of (**a**) CuS and (**b**) N,CuO@CuS core–shell structure. (**c**) EDS image mapping for N,CuO@CuS electrode film. (**d**) XRD and (**e**) Raman spectra of CuS and N,CuO@CuS core–shell structure films. Notably, the three additional XRD peaks (marked with star *) in the spectra are due to the NF substrate.

The material morphologies and compositions of the electrode films were assessed using FESEM and EDS image analysis. Figure 2a,b display the FESEM images of the CuS and N,CuO@CuS electrode films. The CuS electrode film (Figure 2a) reveals an agglomerated, compact spherical morphology with a random, embossed, textured surface topography. The size of these spheres is between 300 and 400 nm. For the N,CuO@CuS electrode film, the material morphology remains almost the same (Figure 2b); however, the surface topography dramatically changes after nitrogenation (inset of Figure 2b). The well-defined flake-like texture on the surface, covering the entirety of the agglomerated CuS spheres, might be due to surface oxidation upon high-temperature heating. These ultrathin nanoflake-like embossed textured shells (i.e., CuO) on the surfaces of interconnected CuS spheres (i.e., core) form a core–shell structure, preventing the degradation of the catalytic activity of the formed CuS material. Further, the incorporated N atom could result in a higher specific surface area and faster electrochemical reaction kinetics. The EDS spectra (Figure S2) and EDS mapping of the N,CuO@CuS electrode film (Figure 2c) reveal the uniform distribution of Cu, S, N, and O elements. Compared to the pure CuS electrode film (Figures S2 and S3), the presence of additional N and O constituents in the nitrogen-treated electrode film confirms the successful attachment of N atoms to the CuS structure and the simultaneous surface transition to the oxide phase during the process, resulting in the formation of a core–shell structure after the nitrogenation process.

### 3.2. Crystallographic Characteristics

The material phase and crystallographic structure were determined through the XRD technique. Figure 2d shows the XRD spectra for the CuS and N,CuO@CuS core–shell structure catalyst electrodes along with the relevant JCPDS patterns. The CuS electrode exhibits Bragg diffractions at 2θ = 29.18°, 31.84°, 38.82°, 48.02°, 53.00°, and 59.32°, which correspond to the (102), (103), (105), (110), (108), and (116) reflection planes of CuS (JCPDS card no. 06-0464), respectively. For the N,CuO@CuS core–shell structure, the XRD spectrum is almost identical to that of the pristine CuS sample; however, the Bragg diffraction peaks are shifted 0.14° towards a higher diffraction angle compared to the pure CuS electrode. Moreover, a simultaneous increase in the full width at half maximum (FWHM) was observed for the diffracted peak, as shown in the inset of Figure 2d (magnified XRD peak associated with the Miller plane (110)). The positive XRD peak shift and broadened FWHM might be a result of the reduced interplanar lattice spacing of the formed core–shell structure compared to pure CuS, due to the successful attachment of N atoms, which have a lower atomic radius (0.075 nm) than those of S (0.102 nm) and Cu (0.138 nm) [15,24]. Further, the structural bonding arrangement in the electrode materials was analyzed using Raman spectral analysis. Figure 2e shows the Raman spectra of the CuS and N,CuO@CuS electrode films. The CuS electrode film demonstrates Raman vibrational peaks at 265 cm^−1^, 474 cm^−1^, and 557 cm^−1^ that correspond to Cu–S vibration, S–S ions’ symmetric stretching at the 4e site, and the phonon longitudinal overtone, respectively [14,19,25]. The Raman spectrum of the N,CuO@CuS core–shell structure is identical to that of the pure CuS but with three additional humps originating from the Ag (299 cm^−1^) and Bg (342 and 591 cm^−1^) modes of the amorphous CuO [26,27]. Moreover, the Raman peaks of the core–shell structure, which are identical to those of CuS, exhibit a blue shift in the peak position due to the increased vibrational frequency arising from the varied surface state of CuS upon nitrogenation.

### 3.3. Chemical State Characteristics

**Figure 3:**
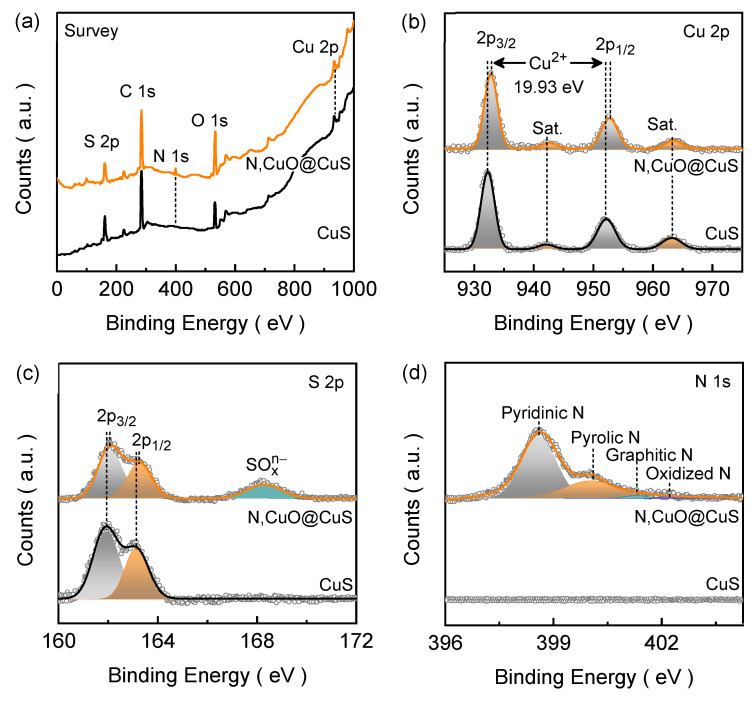
(**a**) XPS survey spectra and high-resolution XPS (**b**) Cu 2p, (**c**) S 2p, and (**d**) N 1s emission spectra for CuS and N,CuO@CuS core–shell structure electrode films. Notably, all of the obtained narrow-range XPS emission spectra are deconvoluted using the Gaussian curve fitting model.

The elemental binding states of the electrode films were examined using XPS spectral analysis. Figure 3a depicts the full-range survey spectra of the CuS and N,CuO@CuS electrode films, which reveal the presence of emission peaks of the constituent elements such as Cu, S, O, and N. Figure 3b shows the well-resolved narrow Cu 2p spectra for the CuS and N,CuO@CuS electrode films. The CuS electrode film demonstrates the typical XPS signals of Cu 2p_3/2_ (932.27 eV) and Cu 2p_1/2_ (952.26 eV), with a binding energy difference of 19.99 eV (~20 eV), confirming the presence of the Cu^2+^ state of Cu in the CuS structure [28]. The remaining two peaks are associated with the satellite peaks of the paramagnetic chemical state of Cu^2+^ located at 942.11 and 962.85 eV (marked as “Sat.” in Figure 3b) [29]. The N,CuO@CuS electrode film also shows similar characteristic peaks in the Cu 2p emission spectra; however, the emission peak positions are slightly blue-shifted compared to those of the pure CuS electrode film, suggesting that the N atom doping in the CuS structure leads to reduced electron density around the neighboring S atoms and its electrons are more tightly bound to the nucleus due to the higher electronegativity and electron trapping capability of nitrogen compared to sulfur atoms [30].

The narrow-range S 2p spectra for the CuS and N,CuO@CuS electrode films are shown in Figure 3c. The S 2p emission spectrum for the CuS electrode film reveals two deconvoluted peaks related to S 2p_3/2_ (161.94 eV) and S 2p_1/2_ (163.13 eV) characteristic emissions [31]. The spin energy separation of 1.19 eV for the characteristic sulfur emission confirms its S^2−^ state in the CuS structure [32]. The spin energies of the distinctive S 2p_3/2_ and S 2p_1/2_ emissions are slightly right-shifted for the N,CuO@CuS electrode film, confirming the successful bonding of N atoms in the CuS lattice. Moreover, an additional wide peak appears in the S 2p spectrum related to SO_x_^n−^ (168.28 eV) emission, and it is associated with surface oxidation [33]. The narrow-range N 1s spectra for the CuS and N,CuO@CuS electrode films are presented in Figure 3d. Undoubtedly, the nitrogen emission peaks appear only for the N,CuO@CuS electrode film. These emission peaks are associated with pyridinic N (i.e., metal nitrogen bonding, 398.60 eV), pyrrolic N (400.05 eV), graphitic N (401.31 eV), and oxidized N (402.23 eV), respectively [34,35]. The pyridinic and pyrrolic N species’ peaks contribute significantly to the spectrum and help to enhance the catalytic active sites and conductivity of the doped material. Nonetheless, the oxidation states of Cu^2+^ and S^2−^ reconfirm the formation of the CuS phase, and the presence of N and SO_x_^n−^ species confirms the successful doping of N atoms into the CuS lattice and the partial surface oxidation of the catalyst structure, as evidenced by the observed surface topological change in the FESEM image (Figure 2b).

### 3.4. Electrochemical OER Performance

**Figure 4:**
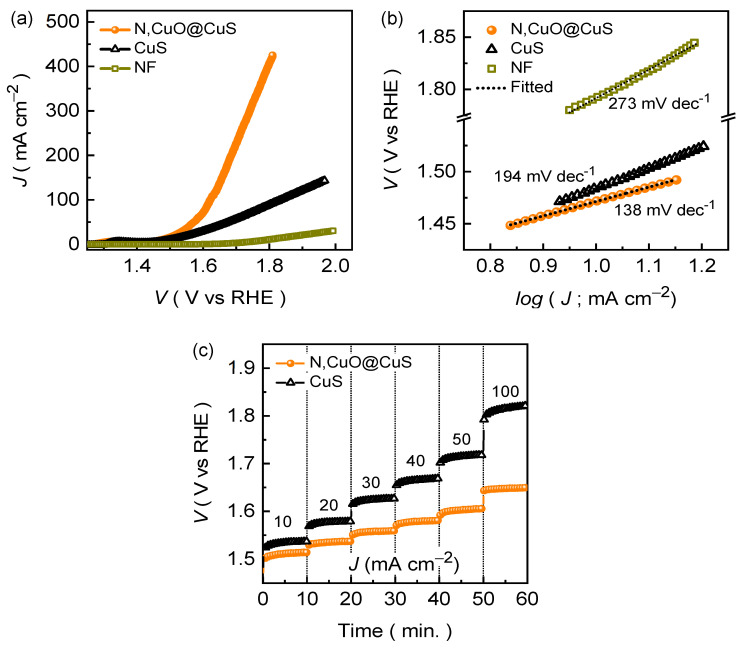
(**a**) LSV curves and (**b**) Tafel plots for the NF, CuS, and N,CuO@CuS core–shell structure electrode films. (**c**) Voltage-step profile for the CuS and N,CuO@CuS catalysts measured at various current densities.

The *JR*-corrected LSV curves for the CuS and N,CuO@CuS electrodes are shown in Figure 4a. For comparison, the LSV curve for the NF substrate was also recorded under the experimental conditions. Clearly, the catalytic activity of the NF substrate is relatively poor (almost inactive) compared to the formed catalysts. The N,CuO@CuS electrode demonstrates good electrochemical activity by attaining a smaller overpotential of 240 mV at a driving current density of 10 mA cm^−2^, whereas the pristine CuS catalyst attains only 268 mV at a similar current density. The change in overpotential is linked to the boosted reaction kinetics, which can be understood by analyzing the Tafel plots. Figure 4b shows the Tafel plots obtained from the LSV curves using the following equation:

*ɳ* = [log (*J*) × *b*] + *a*, (6)

where *b* and *a* are the Tafel slope and constant of the equation, respectively. The NF, CuS, and N,CuO@CuS catalysts demonstrate Tafel slopes of 273, 194, and 138 mV dec^−1^, respectively. The decrease in the Tafel slope value implies that the N,CuO@CuS catalyst has better electrochemical kinetics than the pristine CuS catalyst. This is because the incorporation of nitrogen facilitates a catalytically active surface area (Figure S4) and enhances the material conductivity (Figure S5a), which results in an improved rate of reaction kinetics, as supported by the *ECSA*-corrected LSV curve analysis (Figure S5b) [36].

Nyquist impedance curves (EIS plots) were recorded to help to understand the fundamental electron transfer kinetics of the CuS and N,CuO@CuS core–shell structure catalysts. Figure S5a shows the Nyquist impedance curves for both catalysts along with the tank circuit that was used to fit the semicircle curves (i.e., charge transfer resistance; Rct) [37]. The point at which the semicircles meet the X-axis illustrates the internal resistance of the electrodes (i.e., Rs). This resistance encompasses both the substrate’s intrinsic resistance and the electrolyte’s resistance within the electrochemical system. The N,CuO@CuS possesses a smaller Rct curve compared to the pure CuS catalyst (Table 1), indicating that the conductivity of the catalyst material is increased after the nitrogen treatment, which results in the kinetically more efficient transportation of electrons and ions with the core–shell structure and, thus, results in the superior catalytic activity of the N,CuO@CuS core–shell structure.

**Figure 5:**
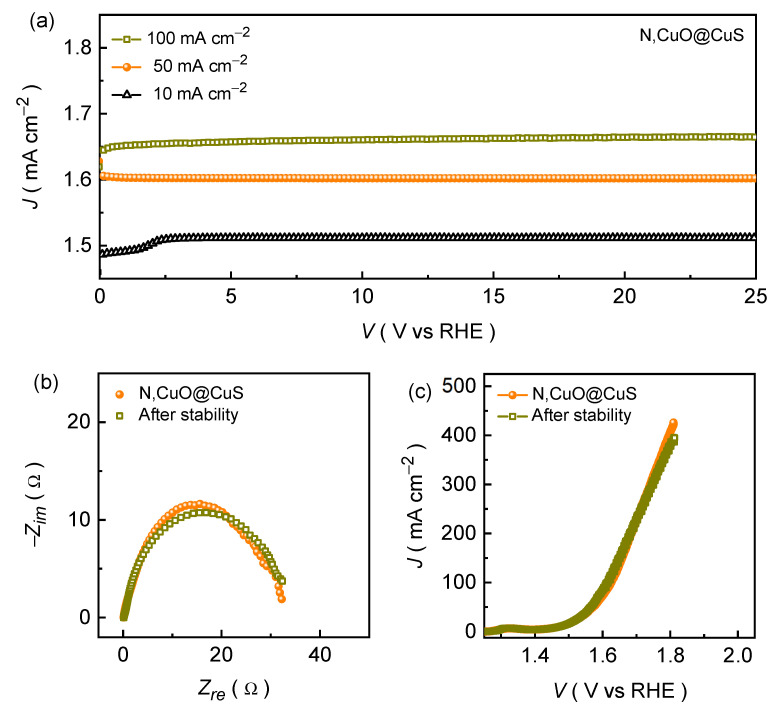
(**a**) Chronopotentiometric long-term OER stability measured up to 25 h at various current densities, post-stability measured (**b**) Nyquist impedance plots, and (**c**) LSV curves for N,CuO@CuS core–shell structure electrode film.

The N,CuO@CuS catalyst further demonstrates excellent electrochemical activity at a higher current density of 20, 30, 40, 50, and 100 mA cm^−2^ by achieving a small overpotential of 282, 307, 328, 341, and 392 mV, respectively. Moreover, the N,CuO@CuS catalyst exhibits a static potential response at various current rates while maintaining the smallest potential response at each driven current density, which is shown in the chronopotentiometric plots (Figure 4c). Moreover, superior endurance during the stability test is another crucial characteristic of a good OER catalyst electrode [23,38]. Figure 5a demonstrates the long-term stability of the formed N,CuO@CuS catalyst, which was recorded at different current rates (10, 50, and 100 mA cm^−2^) in alkaline KOH conditions. The chronopotentiometric curve that was recorded at a current density of 10 mA cm^−2^ demonstrates a rapid increment in the potential during the initial testing, which might have been due to electrochemical activation (i.e., phase transformation), and then the potential remained almost unaffected throughout the OER stability test. Moreover, the N,CuO@CuS catalyst exhibits excellent endurance during the long-term resilience test at a higher current density of 50 and 100 mA cm^−2^. This steady-state potential response within a diverse current density range is a result of the excellent electron and ion transport throughout the chronopotentiometric OER stability test. This analysis is supported by the Nyquist impedance curve (Figure 5b) measured after the long-term chronopotentiometric stability test, which reveals an insignificant change in the Rct (Table 1) value after the OER stability test. Nonetheless, the almost identical LSV (Figure 5c) curves measured after the stability test further attest to the excellent long-term OER performance in an alkaline medium.

To gain further insights into the OER mechanism during stability testing, we consider the chronopotentiometric curve with three distinct potential regions, as illustrated in Figure 5a (black triangle). During the initial testing, the potential increases rapidly up to ~1.488 V (vs. RHE; without *JR* correction), without any trace of gas bubbles on the electrode surface, indicating the non-occurrence of the OER process. The potential gradually increases from 1.488 to 1.512 V (vs. RHE) and bubbles appear simultaneously on the catalyst surface, signifying the onset of the OER process. The change in the potential is associated with phase transformation (i.e., CuO ⇌ Cu(OH)_2_) [38]. Thereafter, a stable voltage of 1.512 V (vs. RHE) is maintained. Meanwhile, a significant number of O_2_ bubbles begin to evolve from the surface at a constant current density of 10 mA cm^−2^ and the evolution of these O_2_ gas bubbles becomes more evident and rapid with increasing current densities of 50 and 100 mA cm^−2^. The ex-situ Raman (Figure S6a) and EDS (Figure S6b) spectra were recorded after the long-term chronopotentiometric test to understand the changes in the chemical bonds and composition of the catalyst. In addition to the characteristic vibrational modes of CuS and CuO, the Raman spectrum of the N,CuO@CuS core–shell structure catalyst measured after the stability test exhibits an additional vibrational peak at ~603 cm^−1^, which is associated with electrocatalytically active Cu species used for water oxidation [27]. Moreover, the EDS spectrum (Figure S6b) reveals slightly increased oxygen content, and this may occur due to the alkaline electrolyte during continuous testing.

## 4. Conclusions

The N,CuO@CuS core–shell structure was successfully synthesized on a 3D porous NF substrate via a mild hydrothermal procedure followed by a nitrogenation process. The surface topography of the pristine CuS was completely altered after the nitrogen treatment and a flake-like textured shell appeared on the surface, which might be beneficial in protecting the formed core–shell-structured catalyst from degradation during electrocatalytic OER stability testing. The N,CuO@CuS catalyst demonstrates excellent OER performance by achieving a small overpotential of 240 and 392 mV compared to the pristine CuS catalyst (268 and 602 mV) at 10 and 100 mA cm^−2^, respectively, with a modest Tafel slope of 138 mV dec^−1^. In addition, the N,CuO@CuS catalyst exhibits an excellent voltage-step profile with a static potential response at various current densities and reveals good endurance over 25 h chronopotentiometric stability testing at 10, 50, and 100 mA cm^−2^. The excellent catalytic OER activity of the core–shell structure is a result of the doped nitrogen atoms, which alter the catalyst’s conductivity and catalytically active sites and result in boosted reaction kinetics favorable for the electrochemical OER process.

## Figures and Tables

**Table 1:** The fitted parameter values of the Nyquist impedance curves for the CuS and N,CuO@CuS core–shell structure catalysts.

Catalyst	Before OER Stability		After OER Stability	
	Rs (Ω)	Rct (Ω)	Rs (Ω)	Rct (Ω)
CuS	0.176	51.09	-	-
N,CuO@CuS	0.109	32.25	0.118	32.82

## Data Availability

The data presented in this study are available on reasonable request from the corresponding author.

## Supplementary Materials

The following supporting information can be downloaded at: https://www.mdpi.com/article/10.3390/nano13243160/s1, Figure S1: OER performance reliability and supporting LSV curves; Figure S2: EDS spectra; Figure S3: EDS image mapping; Figure S4: Non-Faradaic CV curves for the estimation of ECSA; Figure S5: Nyquist impedance curves and ECSA-corrected LSV curves; Figure S6: Post-stability recorded Raman and EDS spectra.
