# Supplementary material for: Nitrogen-Doped CuO@CuS Core–Shell Structure for Highly Efficient Catalytic OER Application
Source: Nanomaterials (Basel). 2023 Dec 17;13(24):3160. doi: 10.3390/nano13243160 (PMC10745488; doi:10.3390/nano13243160)
Supplement: Supplementary file 1 [file nanomaterials-13-03160-s001.zip › nanomaterials-2730989-supplementary.pdf]

Supporting Information

Supporting Figures

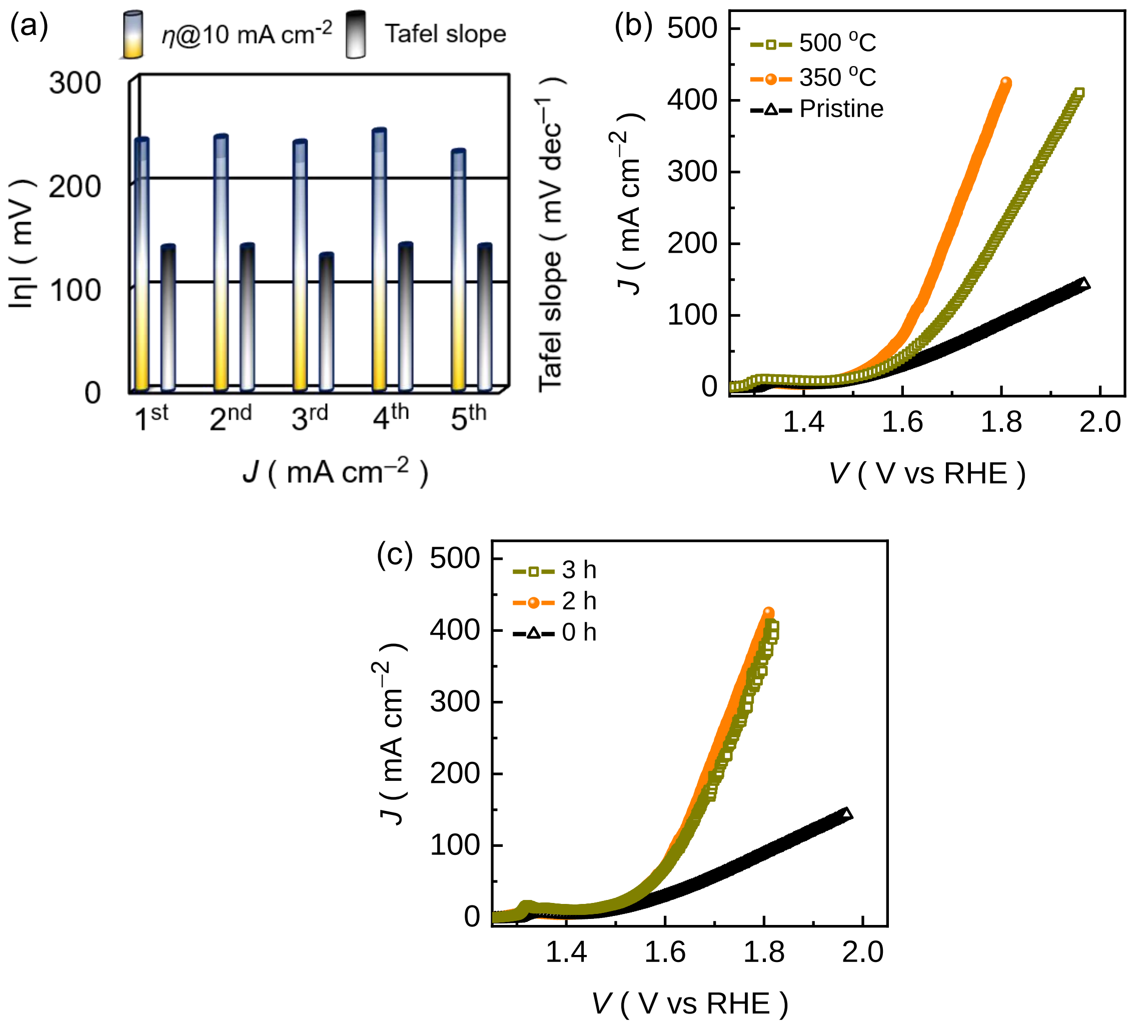

Figure S1. (a) Reliability of the OER activity for the N,CuO@CuS core-shell structure catalyst. (b) Temperature and (c) time dependent LSV curves.

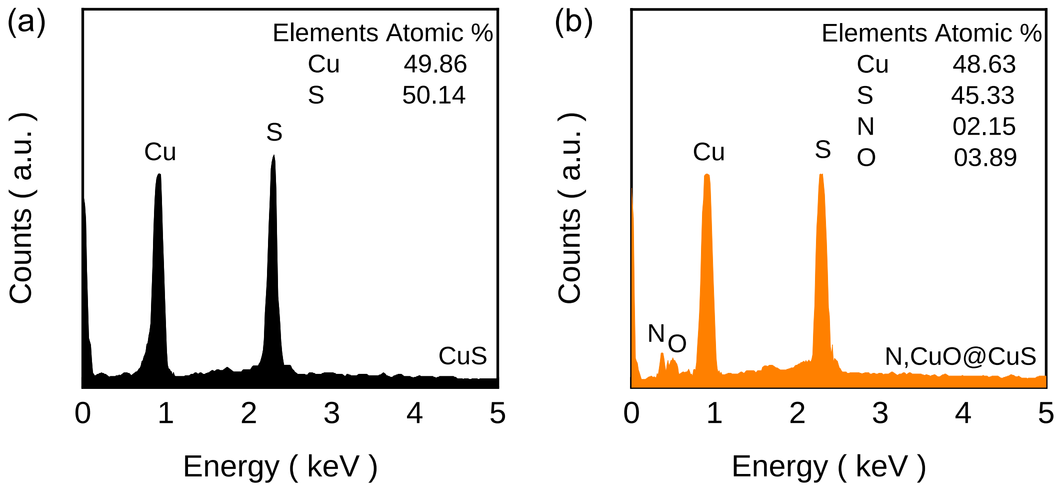

Figure S2. EDS spectra for the (a) CuS and (b) N,CuO@CuS electrode films.

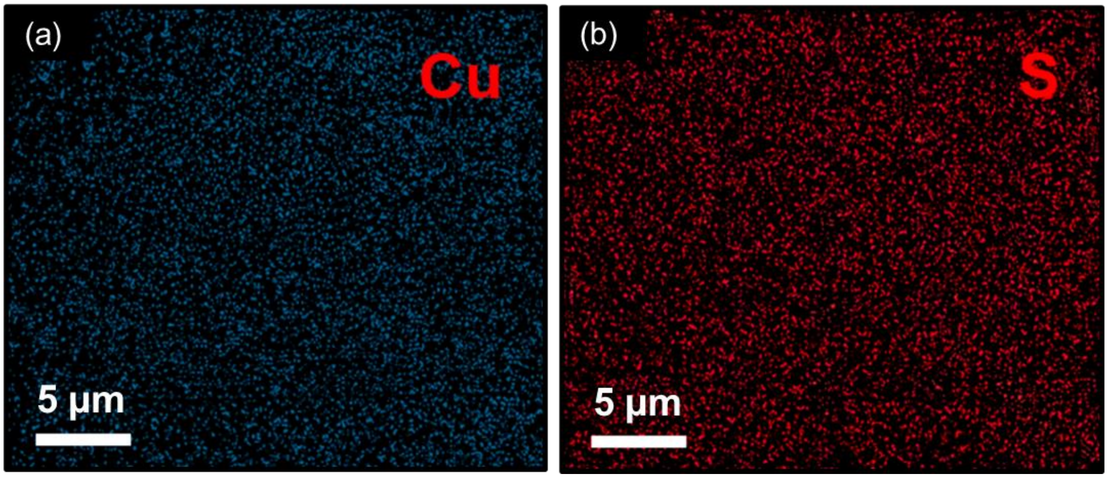

Figure S3. (a) Cu and (b) S constituents EDS image mapping for the pure CuS electrode film.

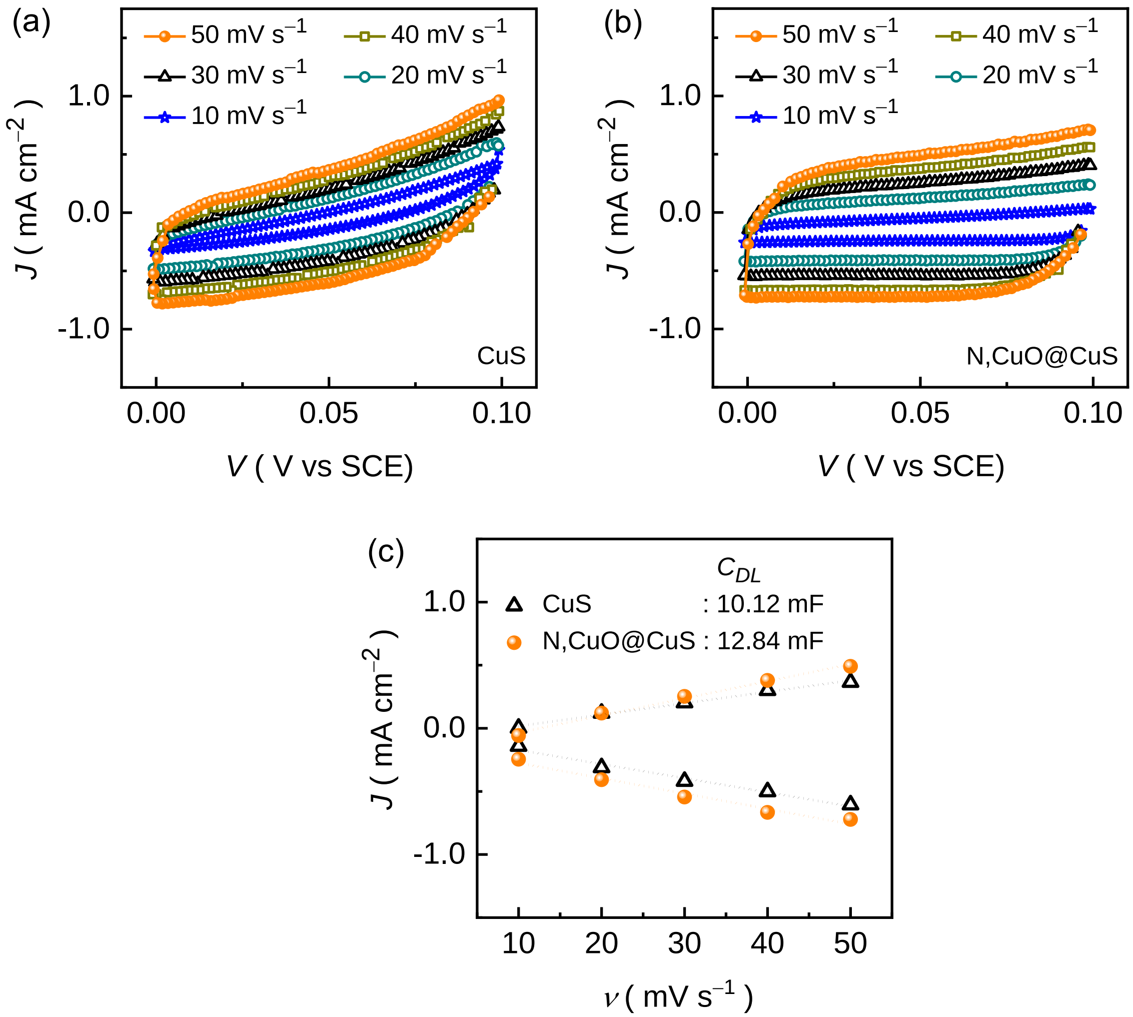

Figure S4. Non-Faradaic CV curves for (a) CuS and (b) N,CuO@CuS catalyst measured at various scan rates. (c) "J_{DL} versus v" plots obtained at 0.05 V (vs. SCE) from non-Faradaic CV curves to estimate the double-layer capacitance and ECSA. Notably, the average magnitude value of C_{DL} (i.e., positive and negative slopes obtained from the anodic and cathodic CV sweeps plots, respectively.) was used to calculate *ECSA*

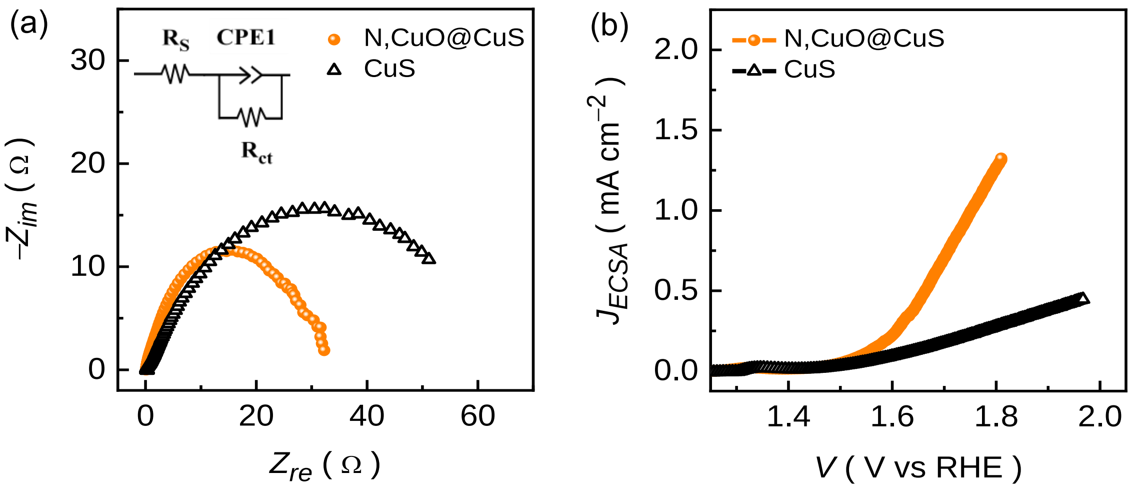

Figure S5. (a) Nyquist impedance spectra and (b) *ECSA*-corrected LSV curves of the CuS and N,CuO@CuS catalysts.

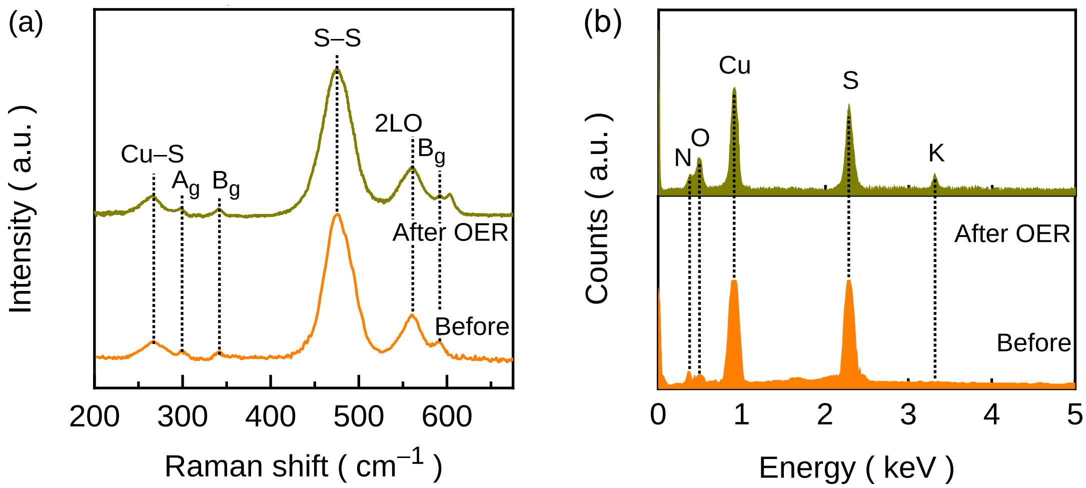

Figure S6. (a) Raman (b) EDS spectra for the CuS and N,CuO@CuS catalysts measured after the long-term chronopotentiometric test.
